# Nobiletin as a Neuroprotectant against NMDA Receptors: An In Silico Approach

**DOI:** 10.3390/pharmaceutics14061123

**Published:** 2022-05-25

**Authors:** Sadaf Jahan, Neeru Singh Redhu, Arif Jamal Siddiqui, Danish Iqbal, Johra Khan, Saeed Banawas, Mohammed Alaidarous, Bader Alshehri, Shabir Ahmad Mir, Mohd Adnan, Aditya Bhushan Pant

**Affiliations:** 1 Department of Medical Laboratory Sciences, College of Applied Medical Sciences, Majmaah University, Al-Majmaah 11952, Saudi Arabia; da.mohammed@mu.edu.sa (D.I.); j.khan@mu.edu.sa (J.K.); s.banawas@mu.edu.sa (S.B.); m.alaidarous@mu.edu.sa (M.A.); b.alshehri@mu.edu.sa (B.A.); s.mir@mu.edu.sa (S.A.M.); 2 Department of Molecular Biology, Biotechnology and Bioinformatics, Chaudhary Charan Singh Haryana Agricultural University, Hisar 125004, Haryana, India; neru.redhu95@gmail.com; 3 Department of Biology, College of Science, University of Hail, P.O. Box 2440, Hail 55476, Saudi Arabia; arifjamal13@gmail.com (A.J.S.); drmohdadnan@gmail.com (M.A.); 4 Department of Biomedical Sciences, Oregon State University, Corvallis, OR 97331, USA; 5 System Toxicology & Health Risk Assessment Group, CSIR-Indian Institute of Toxicology Research (CSIR-IITR), Vishvigyan Bhavan, 31, Mahatma Gandhi Marg, P.O. Box No. 80, Lucknow 226001, Uttar Pradesh, India; ab.abpant@gmail.com

**Keywords:** neurodegenerative disorders, nobiletin, NMDA receptors, ADMET study, neuro-protectant, glutamate receptors

## Abstract

Excitotoxicity is a type of neurodegenerative disorder. It caused by excessive glutamate receptor activation, which leads to neuronal malfunction and fatality. The N-methyl-D-aspartate (NMDA) receptors are found in glutamatergic neurons, and their excessive activation is primarily responsible for excitotoxicity. They are activated by both glutamate binding and postsynaptic depolarization, facilitating Ca^2+^ entry upon activation. Therefore, they are now widely acknowledged as being essential targets for excitotoxicity issues. Molecular docking and molecular dynamics (MD) simulation analyses have demonstrated that nobiletin efficiently targets the binding pocket of the NMDA receptor protein and exhibits stable dynamic behavior at the binding site. In this study, five potential neuroprotectants, nobiletin, silibinin, ononin, ginkgolide B, and epigallocatechin gallate (EGCG), were screened against the glutamate NMDA receptors in humans via computational methods. An in silico ADMET study was also performed, to predict the pharmacokinetics and toxicity profile for the expression of good drug-like behavior and a non-toxic nature. It was revealed that nobiletin fulfills the criteria for all of the drug-likeness rules (Veber, Lipinski, Ghose, Muegge, and Egan) and has neither PAINS nor structural alerts (Brenks). In conclusion, nobiletin demonstrated a possible promising neuroprotectant activities compared to other selected phytochemicals. Further, it can be evaluated in the laboratory for promising therapeutic approaches for in vitro and in vivo studies.

## 1. Introduction

**Figure 1:**
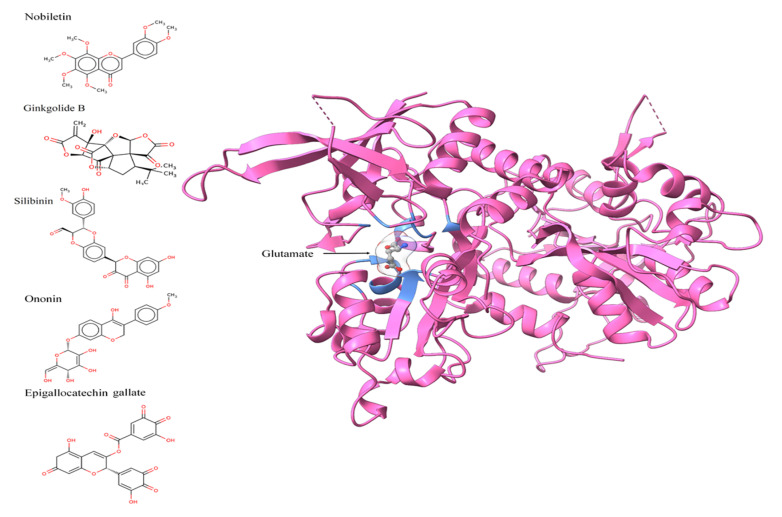
Two-dimensional images of nobiletin, ginkgolide B, silibinin, ononin, and epigallocatechin gallate, and three-dimensional pose of NMDA receptor displaying the glutamate binding and amino acids within <5 angstrom distance, in blue color.

Neurodegenerative disorders are a diverse collection of illnesses with a wide range of clinical manifestations and genetic causes. In developed countries, a changed lifestyle has been accompanied by an increase in the prevalence of neurodegenerative illnesses [1]. Glutamatergic neurons are the brain’s main excitatory system, and they play a key part in a variety of neurophysiological issues. Glutamate is the key neurotransmitter in basic perception and cognition in the brain, and it produces an excitatory response under normal conditions [2,3]. This response is triggered when glutamate interacts with the receptors that make up cation channels. Excessive glutamate receptor activation can cause neuronal malfunction and death, a condition that is known as excitotoxicity [1,4], where there is an excess of glutamate and glutamatergic activity [5]. N-methyl-D-aspartate receptors (NMDARs) are a subclass of glutamate receptors that are activated by glutamate binding and postsynaptic depolarization, and they facilitate Ca^2+^ entry when active [6,7]. NMDAR dysfunction, which can be caused by changes in receptor-channel activity, subunit expression, trafficking, or localization, has been linked to several neurological and psychiatric disorders [8,9]. Synaptic dysfunction is now thought to be linked to many nervous system illnesses. It is becoming increasingly obvious that NMDAR hyperactivity or hypofunction might have negative consequences [3,10]. NMDAR dysfunction can cause central nervous system (CNS) disease in a variety of ways: excessive activation can cause neuronal death, as in stroke and possibly Huntington’s disease, or reduced activity can disrupt the balance of excitation and inhibition in the neural circuitry, affecting CNS functions, as in schizophrenia [11,12]. Additionally, there have been reports of a role of NMDAR in Alzheimer’s disease (AD) and Parkinson’s disease (PD) [11,12,13]. Both excitatory and inhibitory neurons have NMDARs at glutamatergic synapses. Because the excitatory and inhibitory neurons have different and often opposing activities, the functional contributions of these NMDARs are likely to be varied or even opposed. Increased NMDAR function in excitatory neurons, for example, could lead to increases in their synaptic plasticity. However, increased NMDAR function in inhibitory neurons is likely to promote their inhibition (and so reduce excitement), and hence reduce the synaptic plasticity of excitatory neurons [14,15,16]. As a result, changes in NMDAR expression/activity can disrupt the balance between excitation and inhibition and circuit and brain function, depending on their locus. The contributions of NMDARs to CNS illnesses may also be influenced by the subunit makeup and/or the sub-synaptic placement of these receptors at excitatory synapses [17,18,19]. NMDARs are heterotetrametric, with two GluN1 (formerly known as NR1) subunits and two GluN2 subunits (previously known as NR2, encoded by four different genes, GluN2A–D). Variable GluN2A–D subunits in NMDARs have varying electrophysiological and pharmacological properties, and slightly different distribution and expression profiles [20,21,22,23]. Numerous protein interaction and phosphorylation sites can impact upon receptor activation and trafficking in the C-terminal cytoplasmic domains of GluN2A and GluN2B [24,25,26]. Although GluN2A-NMDARs and GluN2B-NMDARs have some common binding partners (such as PSD-95), they have different binding partners for other proteins [20]. The GluN2A-NMDARs bind to Homer and b-Catenin, whereas GluN2B-NMDARs bind to Ca^2+^/calmodulin-dependent protein kinases II (CaMKII) and synaptic Ras GTPase-activating protein (SynGAP). The C-terminal tails of GluN2A and GluN2B may also play a role in GluN2A and GluN2B’s apparent differences in excitotoxicity and neuronal survival. NMDAR activation can either be harmful to neurons or beneficial to their survival and plasticity. NMDARs can produce excitotoxicity when neurons are exposed to glutamate for an extended period. Synaptic NMDAR activity, on the other hand, is required for neuron survival, and inhibiting NMDAR activity in vivo, particularly during development, causes neuronal apoptosis. In some chronic neurodegenerative illnesses, such as Huntington’s disease, NMDAR-mediated excitotoxicity may contribute to neuronal death [26,27]. NMDAR-dependent excitotoxicity appears to be the main cause of neuronal mortality after ischemia or injury in stroke and TBI. NMDAR blockers protect neurons from ischemic cell death in vitro and in vivo. The pathophysiology of neurodegenerative illnesses is heavily influenced by oxidative stress. Evidence suggests that disruptions in systems that use the excitatory amino acid L-glutamate may be at the root of chronic neurodegeneration in AD [14,28,29]. Excitotoxicity caused by excessive NMDAR activation may increase the localized susceptibility of neurons, similar to AD neuropathology, resulting from a change in the regional distribution of NMDA receptor subtypes. NMDA receptor antagonists show promise as a treatment for AD [14,28,30]. Many naturally occurring chemicals have been identified as potential candidates for medication development and management in clinics. Still, numerous phytochemicals are in the queue for research purposes. *Silybum marianum* (milk thistle) silibinin (sylibin) has been shown to have anti-oxidative and anti-inflammatory properties [31]. Neuronal injury, synaptic plasticity impairment, and glutamate cytotoxicity may arise from proinflammatory cytokines activating mitogen-activated protein kinases (JNK and p38). Furthermore, it stimulates NMDA receptors, reducing long-term potentiation (LTP), and contributing to cognitive decline. Ginkgolides affect glutamate transmission, the primary excitatory neurotransmitter in the cortex and hippocampus. Ionotropic receptors and G-protein-coupled metabotropic receptors are the two basic types of glutamate receptors. There are three types of ionotropic receptors, AMPA (α-amino-3-hydroxy-5-methyl-4-isoxazolepropionic acid) receptor; NMDA (N-methyl-D-aspartate) receptor and KA (kinate) receptor. By enabling the flow of ions inside or outside the cell, glutamate binds to receptors and initiates a short-time modulation pathway. On the other hand, activating the metabotropic receptor initiates long-term neural regulation. The passage of calcium ions triggers the secretion of glutamate. According to studies, both NMDAR and AMPAR are inhibited by ginkgolides [32]. Epigallocatechin gallate (EGCG) has been shown to have promising radical scavenging and metal chelation properties, along with the ability to activate and inhibit signaling pathways (MAPK, PKC and PI3K/Akt), enhance antioxidant action (radical scavenging, lipid peroxidation, and the production of endogenous defenses), and modulate cell survival and death genes (anti-apoptotic action), neurite growth, and bioenergetic action (stabilization) [33]. Nobiletin (5,6,7,8,3′,4′-hexamethoxyflavone, NOB) (Figure 1), with an empirical formula of C_21_H_22_O_8_ and a molecular weight of 402.39, is a non-toxic dietary polymethoxyflavone (PMF) that is mostly isolated from citrus fruits. A compound’s bioactivities are determined by its structure and metabolism. Because of its high lipophilic nature and permeability, nobiletin is efficiently absorbed, even without a glycoside moiety, and nobiletin has also been reported as an anti-stress agent [34,35]. Memory and learning are similarly dependent on the NMDA receptor. Nobiletin has been shown to upregulate the mRNA expression of the NMDA receptor subunits NR1, NR2A, and NR2B and c-FOS in PC12 cells [36]. By activating extracellular signal-mediated kinases, nobiletin also improves learning impairment caused by NMDA receptor antagonists. Wet lab research is promising, yet time-consuming. A computational approach is considered to be the easiest, most economical, and best tool for identifying and rapidly selecting phytochemicals against any diseases. Therefore, the authors opted for an *in silico* study for screening herbal compounds against neurological disorders in this research. First, we selected herbal compounds with known anti-oxidative, anti-inflammatory, and anti-neuroprotective activities, and then based it on a 400–500 kDa molecular weight range with a compound that obeyed the Lipinski rule of five. Therefore, five compounds, nobiletin, silibinin, ononin, ginkgolide B, and epigallocatechin gallate were selected for the ‘in silico’ study. We explored the efficacy of the herbal compounds against the NMDA receptors via a computational approach to find the best potential natural compound as a neuroprotective agent.

## 2. Materials and Methods

### 2.1. Sequence Retrieval and Analysis

To identify potentially conserved binding sites in the evolution, which would further help with understanding the structurally conserved binding pocket, protein and gene sequence analyses of the NMDA receptor genes, protein, and isoform were performed. Human N-methyl D-aspartate (NMDA) Glutamate receptor genes (GRIN2A, GRIN2B, GRIN2C, and GRIN2D), protein, and isoform sequences were retrieved from NCBI (http://www.ncbi.nlm.nih.gov/, accessed on 4 October 2021) [37] (Gene ID: 2903, 2904, 2905, and 2906; Accession Nos. (complete CDS): BC117131.1, BC113620.1, BC140801.1, and U77783.1); and UniprotKB (Accession Nos.: Q12879, Q13224, Q14957, and O15399), respectively [38] (Table 1). The aliphatic index, instability index, molecular weight, hydropathicity, and theoretical pI values of UGPase were calculated using ProtParam [39].

### 2.2. Sequence Alignment and Phylogenetic Analysis

Multiple sequence alignments of the genes and protein sequences were performed using muscle, and a phylogenetic tree was generated using the MEGAX tool, using the maximum likelihood and minimum evolution method, with 1000 bootstrapping values and the Tamura-Nei model (for nucleotide sequences), the Jones, Taylor, and Thornton model (for protein sequences), uniform rates among sites, with gaps or missing data treated as partial deletions, and a site coverage cut-off of 95%. The initial tree was obtained using the neighbor-joining method, whereas alternative trees were generated using the Close-Neighbor Interchange method.

### 2.3. Protein Structure, Chemical/Ligand Retrieval, and Analysis

The 3D structure of the GluN1/GluN2A ligand-binding site with a 2.2 Å resolution (PDBID; 5H8F) was retrieved from Protein Databank [38] https://www.rcsb.org/, accessed on 10 November 2021) for docking. The structures of nobiletin (CHEBI ID: 7602), ononin (CHEBI ID: 7775), silibinin (CHEBI ID: 9144), ginkoglide B (CHEBI ID: 5356), and epigallocatechin gallate (CHEBI ID: 4806) were retrieved from the CHEBI database [39] in molfile format (Figure 1). The structures were further optimized and minimized using UCSF ChimeraX, followed by their conversion to pdb format [40]. According to the Lipinski, Ghose, Veber, Egan, and Muegge rules/filters, the molecules were then assessed using SwissADME for drug-likeness [41,42].

### 2.4. Molecular Docking

**Figure 2:**
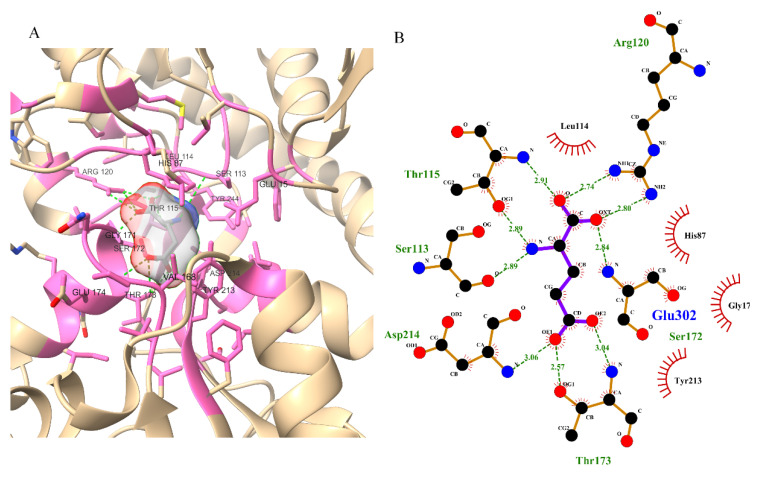
Three-dimensional (**A**) and two-dimensional poses (**B**) of glutamate with the NMDA receptor; glutamate showing hydrogen bonds with Ser113, Thr115, Arg120, Asp214, Ser172, and Thr173; and hydrophobic interactions with His87, Leu114, Gly171, and Tyr213.

**Figure 3:**
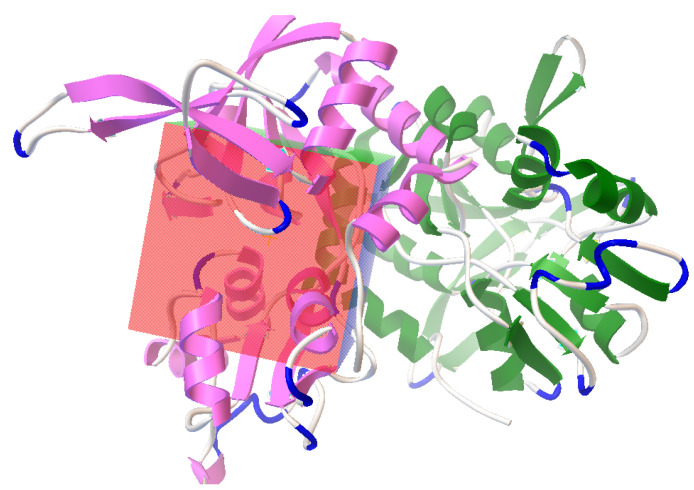
Autodock-generated grid box around glutamate binding site in NMDA receptor for the docking of nobiletin, ginkgolide B, silibinin, ononin, and epigallocatechin gallate molecules.

Auto Dock [43] was used for molecular docking. The pocket containing bound glutamate was selected and retrieved from the protein (Figure 2). Both flexible and rigid docking were performed. A grid box was generated around the active 16.774, −17.218, and −12.686 as the x, y, and z coordinates, respectively (Figure 3). The docking parameters included an external energy grid as 1000, a population size of 250, a maximum number of generations as 27,000, and a maximum number of energy evaluations as 2,500,000 and 50 hybrid GA-LS runs for both flexible and rigid docking [44]. The 50 conformations of the molecules with binding energy and docking, the interaction energy of the ligand, its geometric coordinates, and a summary of the interaction energies, such as grid score, electrostatic energy, and van der Waals forces, were obtained using the genetic algorithm as the docking result. The intermolecular energy and other terms were calculated through the docking software, using the methodology mentioned in the manual. The docking software evaluates the intermolecular energy by combining the ligand and protein in their bound conformation. We selected the top single pose for the top cluster having a maximum number of poses in the docking studies. The top pose from docking was used for MD studies, using GROMACS for checking its stability within the binding pose. Subsequently, the binding poses of each molecule were observed, and their interactions with the protein were characterized [44,45]. The best and most energetically favorable conformations of each molecule were selected, following with the complex containing GluN1/GluN2A bound with each molecule, generated in pdbqt format using AutoDock. The complexes were further converted to the pdb format using pymol, for further analysis [44,46,47].

### 2.5. Molecular Dynamic Simulation

The complex with the lowest binding energy was selected for a molecular simulation, which was performed using GROMACS version 2020.4 developed by the GROMACS development teams at the Royal Institute of Technology and Uppsala University, Sweden. GROMACS is a free, open-source software released under the GNU General Public License (GPL) [48]. Numerous studies have indicated the use of MD studies for the determination of the time-dependent stability of compounds [49,50,51]. A topology file was generated using force field CHARMM27, an all-atom force field with the TIP3P water model [52,53]. The protein complex was placed at the center of the dodecahedron box center, with a distance of 1.0 nm from the box edge. A total of 26029 solvent molecules and 7 Cl^−^ ions were added to the system. We used the steepest descent method for energy minimization. Further, isobaric and isothermal equilibration was performed for 200 picoseconds, using the leap frog algorithm. We used the Parrinello-Rahman barostat method and the V-rescale thermostat method in the MD calculations. The system was thermalized before commencing the production run. The LINCS algorithm was used for bond constraints, while the verlet cutoff scheme was used for neighbor searching. Electrostatic interactions were calculated using the Particle Mesh Ewald method. Van der Waals interactions were computed using the force-switch method. The leap frog integrator was used for calculating equations of motion. Structures for nobiletin and other compounds were parameterized using the swiss param tool. The production run was performed for 100 ns. An MD simulation with parameters such as temperature (300 K), pressure (1.0 bar), and density (1000 kg m^−3^) were stabilized over a set period (100 ns), and executed for 100 ns (i.e., time = nstep*dt, nstep = 5 crore, dt = 0.002 fs) [54,55,56]. The RMSD of the simulated and un-simulated complex were graphically plotted using the grace tool.

### 2.6. Protein–Ligand Interactions and ADMET Analysis

Structural changes and interactions were identified using Ligplot+ [57]. The solvent accessible surface area for residues was calculated using the NACCESS tool [58]. Absorption, distribution, metabolism, excretion and toxicity were predicted using the SwissADME tool and the vNN-ADMET tool [42,59].

## 3. Results

### 3.1. Sequence and Phylogenetic Analysis

NMDA glutamate receptor genes, protein, and isoform sequences were retrieved from NCBI (http://www.ncbi.nlm.nih.gov/), accessed on 21 November 2022. The stability of the NMDA glutamate receptors, i.e., GRIN2A, GRIN2A, GRIN2B, GRIN2C, and GRIN2D, was identified through instability and the aliphatic index score, using ProtParam. The GRAVY score, the aliphatic index and the melting point all point towards the structures of the glutamate receptors (Table S1). The CDS and protein sequences were aligned using the Muscle program from MEGAX (Figures S1 and S2). The estimated half-life of each NMDA protein was 30 h. Similar results among the NMDA glutamate receptors denoted the conservation in their physiochemical properties during evolution. Two conserved regions were predicted in the mature RNA coding sequence, using 0.2 as the maximum allowed entropy of 14 base pairs in length (1866–1879 and 2145–2158 bps). Additionally, two conserved regions were predicted in protein sequences that were 24 (670–693; VWAFFAVIFLASYTANLAAFMIQE) and 15 (760–774; GKLDAFIYDAAVLNY) amino acids in length. The maximum likelihood and minimum evolution method using bootstrapping from the MEGAX tool were employed for building phylogenetic tree using CDS and protein sequences. For both GRIN2A and GRIN2B, closer evolutionary relationships were depicted compared to GRIN2C and GRIN2D. (Figure S3). Through the sequence analysis of the NMDA protein, it can be concluded that the binding site of glutamate is highly conserved during the evolution, and this indicates that it is important for functionality. It can be targeted for therapeutic potential.

### 3.2. Active Site Identification and Ligand Preparation

The 3D structure of the GluN1/GluN2A protein contains glutamate as one of the bound ligands. The active site was determined from bound glutamate. Ten amino acids were determined as the active residues, including H87, S113, L114, T115, R120, G171, S172, T173, Y213, and D214, among others (Figure 2). The surface area and volume of the protein were computed as being 1415.032 and 1241.096, respectively. The flexible residues were H87, T115, S113, L114, R120, S172, T173, Y213, and D214. Optimization and structure minimization were performed using the steepest descent method (100 steepest descent steps and a step size of 0.02 Å) and conjugate gradient (10 conjugate gradient steps and a step size of 0.02 Å). The AMBER ff14SB forcefield assigned partial charges with the gasteiger method. The minimum energies obtained after optimization were 328.62 kJ/mol for nobiletin (CHEBI ID: 7602), 299.18 kJ/mol for ononin (CHEBI ID: 7775), 225.26 kJ/mol for silibinin (CHEBI ID: 9144), 515.44 kJ/mol for ginkgolide B (CHEBI ID: 5356), and 349.33 kJ/mol for epigallocatechin gallate (CHEBI ID: 4806).

### 3.3. Molecular Docking

**Figure 4:**
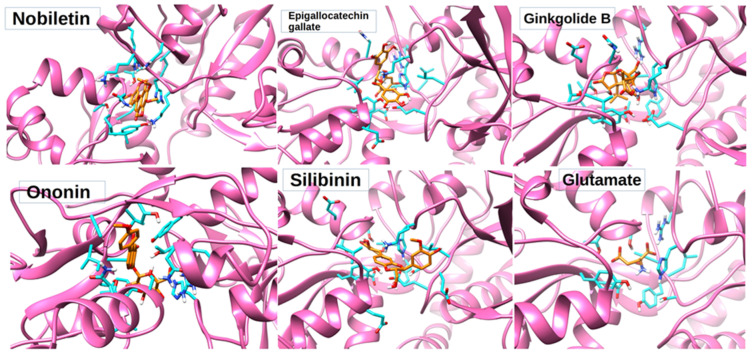
Three-dimensional poses of nobiletin, ginkgolide B, silibinin, ononin, and epigallocatechin gallate docked with NMDA receptor through rigid docking parameters and displaying amino acids within <5 angstrom distance in orange color.

The best poses with the lowest binding energies were selected after rigid docking (Table 2). Rigid docking produced the lowest binding energy of −6.66 KJ/mol for nobiletin, 1.67 KJ/mol for silibinin, −4.37 KJ/mol for ononin, 42.73 KJ/mol for ginkoglide B, and −2.87 KJ/mol for epigallocatechin gallate (Table 2). The intermolecular energy was calculated to be −8.75 KJ/mol for nobiletin, −0.42 KJ/mol for silibinin, −7.35 KJ/mol for ononin, 42.43 KJ/mol for ginkoglide B, and −4.95 KJ/mol for epigallocatechin gallate. The lowest binding energy (−6.66 KJ/mol) and intermolecular energy (−8.75 KJ/mol), as well as the van der Waals and hydrogen bond desolvation energy (−2.79 KJ/mol) of nobiletin obtained from rigid docking was the highest among all of the compounds. All the molecules were observed to be binding to the same binding pocket as glutamate (Figure 4). Since rigid docking is unable to address the stability of the ligand within the binding pocket, these molecules were subjected to MD studies to check their time-dependent stability.

### 3.4. Molecular Dynamics Simulation

**Figure 5:**
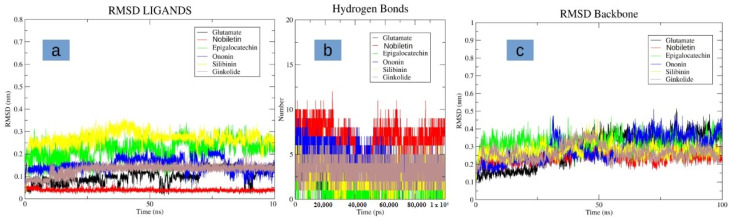
Displaying the molecular dynamics (MD) simulation. (**a**) RMS fluctuation of different and glutamate in the binding pocket of NMDA receptor (**b**) Hydrogen bond plots of the different ligands and glutamate with the NMDA receptor (**c**) RMSD of the protein backbone of the NMDA receptor when bound with different ligands and glutamate.

After conducting the docking studies, we further performed MD studies on these molecules to check their stability in complex with the NMDA protein. Interestingly, nobiletin indicated a high degree of stability, and was observed to be bound to the same binding pocket with minor structural deviations (Figure 5a). It was observed to be highly stable, with a low RMSD of less than 1 Å. Additionally, it indicated the highest number for hydrogen bond formation during simulation, as compared to the glutamate molecule, as well as other selected molecules (Figure 5b). The compounds, namely epigallocatechin gallate, ononin, and silibinin were found to be unstable, and indicated higher deviations of up to 3 Å (Figure 5a). Molecule ginkolide was also observed to be stable in its binding pocket after initial slight deviations in its structure. All of the molecules had, on an average, three to five hydrogen bonds, and were comparable to the glutamate molecule in terms of hydrogen bonds (Figure 5b). The protein backbone was also observed to be stable while bound in complex with the nobiletin, and indicated a low RMSD of 3 Å. Compared to the control, the deviations were observed to be more stable for the protein backbone (Figure 5c). For other compounds, deviations were mostly similar to the glutamate-bound protein backbone. In summary, the ligand nobiletin was observed to be highly stable in its binding pocket, as compared to other compounds, as well as the glutamate molecule. This high degree of stability may be attributed to more hydrogen bond interactions with the receptor.

### 3.5. Protein–Ligand Interaction Analysis

The interacting amino acids was identified using Ligplot+ for every complex obtained from the rigid docking molecules (Table 3). The three-dimensional poses of nobiletin, ginkgolide B, silibinin, ononin, and epigallocatechin gallate docked with the NMDA receptor have been summarized in Figure 4. After performing the interaction analysis for various non-covalent interactions, it was observed that the residues involved in hydrogen bond formation were Glu14, Lys86, His87, Gly88, Thr115, Arg120, Asn170, and Asn192 (Table 3). A significant change in a solvent-accessible area of amino acids constituting the catalytic pocket H87, S113, L114, T115, R120, G171, S172, T173, Y213, and D214 etc. (Table 3) was observed. The change in the solvent accessible area corresponds to an increase of 290.943 Å for epigallocatechin gallate, 169.565 Å for ginkgolide B, 151.406 Å for ononin, and 118.606 Å for silibinin, while a decrease of −25.084 Å was observed for nobiletin. This might also coincide with the change in the biological activity after binding with nobiletin.

### 3.6. ADME(T) Analysis

Swiss-ADME predicted epigallocatechin gallate and ginkgolide B as being very soluble and soluble, while nobiletin and silibinin were moderately soluble in aqueous solution when using the ESOL [60] and Ali [61] methods. In contrast, ononin was predicted as being soluble via ESOL, but only moderately soluble via the Ali method (Table 4). Nobiletin, silibinin, ginkgolide B, and ononin were predicted to have high gastrointestinal absorption with a bioavailability score of 0.55; this was 0.11 for epigallocatechin gallate. All five nobiletin, silibinin, ginkgolide B, epigallocatechin gallate, and ononin compounds showed a low degree of skin permeation, with a score ranging from −6.62 cm/s to −10.00 cm/s. Both Swiss-ADME and vNN-ADMET predicted all five of the compounds as not being inhibitors of CYP2D6. However, conflicting results were obtained for its properties, such as a P-gp substrate, a CYP1A2 inhibitor, a CYP2C19 inhibitor, a CYP2C9 inhibitor, and a CYP3A4 inhibitor (Table 5). It was also predicted that nobiletin, silibinin, and ononin do not belong to Pan-assay interference compounds (PAINS), which are usually responsible for producing a false-positive reading by mimicking the drug response. However, a potential pain response was predicted for ginkgolide B and epigallocatechin gallate [62]. Another method of finding structural alerts in medicinal chemistry is brenk, which predicted alerts for every compound except nobiletin. Swiss-ADME also predicted their synthetic accessibilities, with scores ranging from 3.90 for nobiletin, to 4.65, 4.80, 4.81, and 6.80, respectively, for silbinin, ononin, epigallocatechin gallate, and ginkgolide B (here, 1 is very easy and 10 is very difficult).

**Figure 6:**
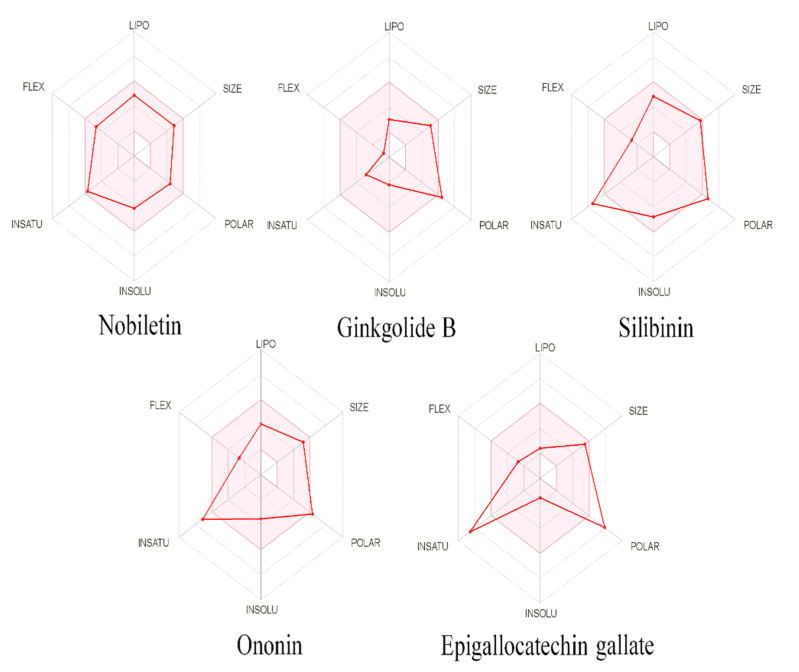
Physiochemical space for oral bioavailability, the colored zone is showing the allowed zone, and the red line showing the predicted natures of nobiletin, ginkgolide B, silibinin, ononin, epigallocatechin gallate.

Drug-likeness was predicted for all five compounds, with the Lipinski filter predicting all five as being drug-like, whereas according to the Ghose filter, epigallocatechin gallate, silibinin, ononin and nobiletin were predicted; according to veber, nobiletin, and ononin were predicted; according to Egan, only nobiletin was predicted, and according to Muegge, ginkgolide B, silibinin, ononin, and nobiletin fit the criteria. Only nobiletin was predicted to have drug-like properties, with no violation of any of the filters among the five compounds. The oral bioavailability was also predicted using SwissADME in polygon with six parameters, i.e., FLEX, LIPO, INSATU, INSOLU, SIZE, and POLAR, representing flexibility lipophilicity, saturation, solubility, and polarity, respectively, of the compounds. The pink shaded area in the polygon represents the optimal range of the six properties, while red lines represent each compound’s predicted physicochemical properties (Figure 6). vNN-ADMET predicted nobiletin as not being cytotoxic, being non-mutagenic, and being both a substrate and an inhibitor of P-gp; silibinin was not an inhibitor of CYP3A4 and CYP2D6, but was a P-gp inhibitor, as well as for CYP2C19 and CYP2C9; no high-confidence predictions were performed for the rest of the compounds. The maximum recommended therapeutic dose was predicted via vNN-ADMET as being 1628 mg/day for nobiletin and 463 mg/day for silibinin.

## 4. Discussion

In the mammalian central nervous system, glutamatergic mechanisms are involved in learning, memory, and cellular plasticity; imbalances in this mechanism have been implicated in several neurodegenerative diseases, including schizophrenia, PD, and AD. Recent research has identified nobiletin as being a potential neuroprotectant [35]. This potential was explored through computational techniques such as docking and molecular dynamics simulation. Both excitatory and inhibitory neurons have NMDARs at glutamatergic synapses. Five compounds, nobiletin, silibinin, ononin, ginkgolide B, and epigallocatechin gallate, were selected for our ‘in silico’ research study. NMDARs are required for neuronal function in physiological processes, and they are involved in excitotoxicity, which leads to neuronal death following an ischemic stroke. Because of low drug tolerance and a narrow therapeutic window, the early treatment of NMDAR blockage with antagonists has not resulted in any successful clinical neuroprotective therapy. NMDAR antagonism can abolish survival signals and disrupt neuronal function, resulting in substantial side effects, since NMDAR has a dual role in life and death signaling in neurons. As a result, it is ideal to target only the NMDARs that cause death, while leaving the pro-survival pathways unaffected. Additionally, because these new prospective medicines target NMDAR’s downstream pathways, they may have a longer therapeutic window [60]. NMDAR’s antagonist has previously been used for treating AD. Compared to glutamate, nobiletin was found to more stable in the binding pocket of the NMDAR. Further, the hydrogen bond plot indicated a higher frequency of bond formation by the compound nobiletin as compared to glutamate, which may be attributed to its higher stability and potency in the binding pockets of NMDARs. Further, in our studies, the nobiletin molecule indicated that its ADMET profile, along with its physiochemical, drug-like nature and properties of medicinal chemistry friendliness, make it a good molecule in terms of its pharmacokinetics and pharmacodynamics parameters. Based on solvent accessible surface area calculations, it was concluded that by blocking the catalytic site entrance, nobiletin could act as an antagonist to hyperactivity caused by glutamate binding to the NMDA receptor. Finally, this study has shown that nobiletin is a potential drug against NMDA hypertoxicity. Therefore, nobiletin can be the best possible natural compound for treating numerous neurological disorders that are caused by NMDA hypertoxicity, including AD, PD, and schizophrenia, and it may act as a potential therapeutic agent for chemotherapeutic treatment.

## Figures and Tables

**Table 1:** The Human N-methyl D-aspartate (NMDA) Glutamate receptor sequences were obtained from the NCBI Refseq nucleotide and protein database and their isoforms.

Name of Gene	Refseq Nucleotide Accession No.	Isoforms	Protein Encoded	UniProt Accession No.	Isoforms (at SwissProt)
GRIN2A	BC117131.1	3	GLUN2A	Q12879	3
GRIN2B	BC113620.1	1	GLUN2B	Q13224	1
GRIN2C	BC140801.1	2	GLUN2C	Q14957	2
GRIN2D	U77783.1	1	GLUN2D	O15399	1

**Table 2:** Docking scores of nobiletin, ginkgolide B, silibinin, ononin, and epigallocatechin gallate poses with NMDA receptor after rigid docking, as mentioned below.

	Rigid Docking				
	Epigallocatechin Gallate	Ginkgolide B	Nobiletin	Ononin	Silibinin
Binding energy	−2.87	42.73	−6.66	−4.37	1.67
Ligand efficiency	−0.09	1.42	−0.23	−0.14	0.05
Intermolecular energy	−4.95	42.43	−8.75	−7.35	−0.42
Vdw_hb_desolvation energy	−4.73	42.7	−8.39	−6.95	0.21
Electrostatic energy	−0.22	−0.26	−0.36	−0.38	0.21
Total internal energy	−2.39	−0.14	−1.21	−2.83	1.34
Torsional energy	2.09	0.3	2.09	2.98	2.09
Unbound energy	−2.39	0.14	−1.21	−2.83	1.34
refRMS	25.37	27.76	7.4	7.43	11.15

**Table 3:** Amino acid residues are involved in hydrogen bonding and hydrophobic interactions, and subsequent change in the solvent-accessible area after rigid docking.

Name of Compounds	Name of Residues
Epigallocatechin gallate	Leu13, Glu14, Glu15, Gly85, Lys86, His87, Gly88, Lys89, Asn96, Ser113, Thr115, Arg120, Val168, Pro169, Asn170, Gly171, Ser172, Thr173, Lys194, Gly195, Val196, Glu197, Tyr213, Asp214, Val217, Tyr244
Ginkgolide B	Leu13, Glu14, Glu15, Lys86, His87, Ser113, Thr115, Arg120, Thr133, Gly134, Ile135, Val168, Pro169, Asn170, Gly171, Ser172, Thr173, Asn176, Gly195, Val196, Tyr213, Asp214, Val217, Thr242, Tyr244
Nobiletin	Glu15, Gly85, Lys86, His87, Gly88, Lys89, Asn96, Ser113, Thr115, Arg120, Thr133, Gly134, Ile135, Val168, Pro169, Asn170, Gly171, Ser172, Thr173, Lys194, Tyr213, Asp214, Val217, Tyr244
Ononin	Glu15 Gly85, Lys86, His87, Gly88, Lys89, Asn96, Ser113, Thr115, Arg120, Thr133, Gly134, Ile135, Val168, Pro169, Asn170, Gly171, Ser172, Thr173, Lys194, Tyr213, Asp214, Tyr244
Silibinin	Leu13, Glu14, Glu15, Val82, Thr83, Gly85, Lys86, His87, Val168, Pro169, Asn170, Asn192, Gln193, Lys194, Gly195, Val196, Glu197, Asp198, Tyr213

**Table 4:** Physiochemical, lipophilicity, water-solubility, drug likeliness, and medicinal chemistry properties of nobiletin, ginkgolide B, silibinin, ononin, and epigallocatechin gallate.

	Water Solubility (Log S and Class)		Bioavailability Score	Medicinal Chemistry		
	ESOL Method	Ali Method		Synthetic Accessibility	PAINS	Brenk
Nobiletin	−4.18 Moderately soluble	−4.47 Moderately soluble	0.55	3.90	0 alert	0 alert
Ginkgolide B	−2.22 Soluble	−2.29 Soluble	0.55	6.18	0 alert	3 alerts: diketo_group, michael_acceptor_1, more_than_2_esters
Epigallocatechin gallate	−1.55 Very soluble	−1.99 Very soluble	0.11	4.81	2 alerts: imine_one_A, quinone_D	2 alerts: chinone_2, diketo_group
Silibinin	−4.81 Moderately soluble	−5.78 Moderately soluble	0.55	4.65	1 alert: imine_one_A	2 alerts: aldehyde, diketo_group
Ononin	−3.53 Soluble	−4.11 Moderately soluble	0.55	4.80	0 alert	2 alerts: acyclic_C=C-O, stilbene

**Table 5:** Pharmacokinetics of nobiletin, ginkgolide B, silibinin, ononin, and epigallocatechin gallate.

	GI Absorption	P-gp Substrate		CYP1A2 Inhibitor		CYP2C19 Inhibitor		CYP2C9 Inhibitor		CYP2D6 Inhibitor		CYP3A4 Inhibitor		Log Kp (Skin Permeation) (cm/s)
	Swiss ADME	Swiss ADME	vNN- ADMET	Swiss ADME	vNN- ADMET	Swiss ADME	vNN- ADMET	Swiss ADME	vNN- ADMET	Swiss ADME	vNN- ADMET	Swiss ADME	vNN- ADMET	Swiss ADME
Nobiletin	High	No	Yes	Yes	No	No	Yes	Yes	No	No	No	Yes	No	−6.62
Silibinin	Low	No	No	No	No	No	No	Yes	Yes	No	No	Yes	Yes	−7.10
Ononin	Low	Yes	No	No	No	No	No	No	No	No	No	No	No	−7.78
Ginkgolide B	Low	Yes	Yes	No	No	No	No	No	No	No	No	No	No	−9.02
Epigallocatechin gallate	Low	No	Yes	No	No	No	No	No	No	No	No	No	No	−10.00

## Data Availability

The original contributions presented in the study are included in the article/Supplementary Materials.

## Supplementary Materials

The following supporting information can be downloaded at: https://www.mdpi.com/article/10.3390/pharmaceutics14061123/s1, Table S1: The analysis of NMDA proteins and their isoforms; Figure S1: Multiple sequence alignment mRNA/coding sequence of GRIN2A, GRIN2B, GRIN2C AND GRIND2D genes; Figure S2: Multiple sequence alignment protein sequences are encoded by GRIN2A, GRIN2B, GRIN2C, and GRIND2D genes; Figure S3: Phylogenetics tree created among (a) nucleotide sequence (b) protein sequence.

## References

[1] Dong X.X., Wang Y., Qin Z.H. (2009). Molecular Mechanisms of Excitotoxicity and Their Relevance to Pathogenesis of Neurodegenerative Diseases. Acta Pharmacol. Sin..

[2] Reiner A., Levitz J. (2018). Glutamatergic Signaling in the Central Nervous System: Ionotropic and Metabotropic Receptors in Concert. Neuron.

[3] Fan X., Jin W.Y., Wang Y.T. (2014). The NMDA Receptor Complex: A Multifunctional Machine at the Glutamatergic Synapse. Front. Cell. Neurosci..

[4] Ince P.G., Eggett C.J., Shaw P.J. (1997). The Role of Excitotoxicity in Neurological Disease. Rev. Contemp. Pharmacother..

[5] Kirmani B.F., Shapiro L.A., Shetty A.K. (2021). Neurological and Neurodegenerative Disorders: Novel Concepts and Treatment. Aging Dis..

[6] Tottori T., Fujii M., Kuroda S. (2019). NMDAR-Mediated Ca^2+^ Increase Shows Robust Information Transfer in Dendritic Spines. Biophys. J..

[7] Tachibana N., Kinoshita M., Saito Y., Ikeda S. (2013). Identification of the N-Methyl-D-Aspartate Receptor (NMDAR)- Related Epitope, NR2B, in the Normal Human Ovary: Implication for the Pathogenesis of Anti-NMDAR Encephalitis. Tohoku J. Exp. Med..

[8] Aloisi E., le Corf K., Dupuis J., Zhang P., Ginger M., Labrousse V., Spatuzza M., Georg Haberl M., Costa L., Shigemoto R. (2017). Altered Surface MGluR5 Dynamics Provoke Synaptic NMDAR Dysfunction and Cognitive Defects in Fmr1 Knockout Mice. Nat. Commun..

[9] Bacq A., Astori S., Gebara E., Tang W., Silva B.A., Sanchez-Mut J., Grosse J., Guillot de Suduiraut I., Zanoletti O., Maclachlan C. (2020). Amygdala GluN2B-NMDAR Dysfunction Is Critical in Abnormal Aggression of Neurodevelopmental Origin Induced by St8sia2 Deficiency. Mol. Psychiatry.

[10] Haroon E., Miller A.H., Sanacora G. (2017). Inflammation, Glutamate, and Glia: A Trio of Trouble in Mood Disorders. Neuropsychopharmacology.

[11] Tikhonova I.G., Baskin I.I., Palyulin V.A., Zefirov N.S., Bachurin S.O. (2002). Structural basis for understanding structure-activity relationships for the glutamate binding site of the NMDA receptor. J. Med. Chem..

[12] Breijyeh Z., Karaman R. (2020). Comprehensive Review on Alzheimer’s Disease: Causes and Treatment. Molecules.

[13] Zhang Z., Zhang S., Fu P., Zhang Z., Lin K., Ko J.K., Yung K.K. (2019). Roles of Glutamate Receptors in Parkinson’s Disease. Int. J. Mol. Sci..

[14] Bardaweel S.K., Alzweiri M., Ishaqat A.A. (2014). D-Serine in Neurobiology: CNS Neurotransmission and Neuromodulation. Can. J. Neurol. Sci..

[15] Parsons M.P., Raymond L.A. (2014). Extrasynaptic NMDA Receptor Involvement in Central Nervous System Disorders. Neuron.

[16] Franchini L., Carrano N., di Luca M., Gardoni F. (2020). Synaptic GluN2A-Containing NMDA Receptors: From Physiology to Pathological Synaptic Plasticity. Int. J. Mol. Sci..

[17] Barnes J.R., Mukherjee B., Rogers B.C., Nafar F., Gosse M., Parsons M.P. (2020). The Relationship between Glutamate Dynamics and Activity-Dependent Synaptic Plasticity. J. Neurosci..

[18] Bozic M., Valdivielso J.M. (2015). The Potential of Targeting NMDA Receptors Outside the CNS. Expert Opin. Ther. Targets.

[19] Pérez-Otaño I., Larsen R.S., Wesseling J.F. (2016). Emerging Roles of GluN3-Containing NMDA Receptors in the CNS. Nat. Rev. Neurosci..

[20] Zhou Q., Sheng M. (2013). NMDA Receptors in Nervous System Diseases. Neuropharmacology.

[21] Salussolia C.L., Prodromou M.L., Borker P., Wollmuth L.P. (2011). Arrangement of Subunits in Functional NMDA Receptors. J. Neurosci..

[22] Zhu S., Stein R.A., Yoshioka C., Lee C.H., Goehring A., McHaourab H.S., Gouaux E. (2016). Mechanism of NMDA Receptor Inhibition and Activation. Cell.

[23] Hansen K.B., Yi F., Perszyk R.E., Furukawa H., Wollmuth L.P., Gibb A.J., Traynelis S.F. (2018). Structure, Function, and Allosteric Modulation of NMDA Receptors. J. Gen. Physiol..

[24] Kalia L.V., Kalia S.K., Salter M.W. (2008). NMDA Receptors in Clinical Neurology: Excitatory Times Ahead. Lancet Neurol..

[25] Salter M.W., Kalia L.V. (2004). SRC Kinases: A Hub for NMDA Receptor Regulation. Nat. Rev. Neurosci..

[26] Fan M.M.Y., Raymond L.A. (2007). N-Methyl-d-Aspartate (NMDA) Receptor Function and Excitotoxicity in Huntington’s Disease. Prog. Neurobiol..

[27] Wesseling J.F., Pérez-Otaño I. (2015). Modulation of GluN3A Expression in Huntington Disease a New N-Methyl-D-Aspartate Receptor-Based Therapeutic Approach?. JAMA Neurol..

[28] Danysz W., Parsons C.G. (2012). Alzheimer’s Disease, β-Amyloid, Glutamate, NMDA Receptors and Memantine-Searching for the Connections. Br. J. Pharmacol..

[29] Liu J., Chang L., Song Y., Li H., Wu Y. (2019). The Role of NMDA Receptors in Alzheimer’s Disease. Front. Neurosci..

[30] Wang R., Reddy P.H. (2017). Role of Glutamate and NMDA Receptors in Alzheimer’s Disease. J. Alzheimer’s Dis..

[31] Jin G., Bai D., Yin S., Yang Z., Zou D., Zhang Z., Li X., Sun Y., Zhu Q. (2016). Silibinin Rescues Learning and Memory Deficits by Attenuating Microglia Activation and Preventing Neuroinflammatory Reactions in SAMP8 Mice. Neurosci. Lett..

[32] Bai S., Zhang X., Chen Z., Wang W., Hu Q., Liang Z., Shen P., Gui S., Zeng L., Liu Z. (2017). Insight into the Metabolic Mechanism of Diterpene Ginkgolides on Antidepressant Effects for Attenuating Behavioural Deficits Compared with Venlafaxine. Sci. Rep..

[33] Spanaki C., Zaganas I., Kounoupa Z., Plaitakis A. (2012). The Complex Regulation of Human Glud1 and Glud2 Glutamate Dehydrogenases and Its Implications in Nerve Tissue Biology. Neurochem. Int..

[34] Huang H., Li L., Shi W., Liu H., Yang J., Yuan X., Wu L. (2016). The Multifunctional Effects of Nobiletin and Its Metabolites In Vivo and In Vitro. Evid.-Based Complement. Altern. Med..

[35] Jahan S., Ansari U.A., Siddiqui A.J., Iqbal D., Khan J., Banawas S., Alshehri B., Alshahrani M.M., Alsagaby S.A., Redhu N.S. (2022). Nobiletin Ameliorates Cellular Damage and Stress Response and Restores Neuronal Identity Altered by Sodium Arsenate Exposure in Human iPSCs-Derived hNPCs. Pharmaceuticals.

[36] Liu Z., Zhao W., Xu T., Pei D., Peng Y. (2010). Alterations of NMDA Receptor Subunits NR1, NR2A and NR2B MRNA Expression and Their Relationship to Apoptosis following Transient Forebrain Ischemia. Brain Res..

[37] Arnemann J. (2018). NCBI. Lexikon der Medizinischen Laboratoriumsdiagnostik.

[38] Apweiler R., Bairoch A., Wu C.H., Barker W.C., Boeckmann B., Ferro S., Gasteiger E., Huang H., Lopez R., Magrane M. (2004). UniProt: The Universal Protein Knowledgebase. Nucleic Acids Res..

[39] Garg V.K., Avashthi H., Tiwari A., Jain A., Ramkete P.W., Kayastha A.M., Singh K. (2016). MFPPI-Multi FASTA ProtParam Interface. Bioinformation.

[40] Kumar S., Stecher G., Li M., Knyaz C., Tamura K. (2018). MEGA X: Molecular Evolutionary Genetics Analysis across Computing Platforms. Mol. Biol. Evol..

[41] Berman H.M., Westbrook J., Feng Z., Gilliland G., Bhat T.N., Weissig H., Shindyalov I.N., Bourne P.E. (2000). The Protein Data Bank. Nucleic Acids Res..

[42] Degtyarenko K., de matos P., Ennis M., Hastings J., Zbinden M., Mcnaught A., Alcántara R., Darsow M., Guedj M., Ashburner M. (2008). ChEBI: A Database and Ontology for Chemical Entities of Biological Interest. Nucleic Acids Res..

[43] Pettersen E.F., Goddard T.D., Huang C.C., Meng E.C., Couch G.S., Croll T.I., Morris J.H., Ferrin T.E. (2021). UCSF ChimeraX: Structure Visualization for Researchers, Educators, and Developers. Protein Sci..

[44] Barret R. (2018). Lipinski’s Rule of Five. Medicinal Chemistry.

[45] Daina A., Michielin O., Zoete V. (2017). SwissADME: A Free Web Tool to Evaluate Pharmacokinetics, Drug-Likeness and Medicinal Chemistry Friendliness of Small Molecules. Sci. Rep..

[46] Morris G.M., Huey R., Lindstrom W., Sanner M.F., Belew R.K., Goodsell D.S., Olson A.J. (2009). AutoDock4 and AutoDockTools4: Automated docking with selective receptor flexibility. J. Comput. Chem..

[47] Seeliger D., de Groot B.L. (2010). Ligand Docking and Binding Site Analysis with PyMOL and Autodock/Vina. J. Comput.-Aided Mol. Des..

[48] Cosconati S., Forli S., Perryman A.L., Harris R., Goodsell D.S., Olson A.J. (2010). Virtual Screening with AutoDock: Theory and Practice. Expert Opin. Drug Discov..

[49] Sharma T., Abohashrh M., Baig M.H., Baig M.H., Dong J.-J., Alam M.M., Ahmad I., Irfan S. (2021). Screening of drug databank against WT and mutant main protease of SARS-CoV-2: Towards finding potential compound for repurposing against COVID-19. Saudi J. Biol. Sci..

[50] Sharma T., Siddiqi M.I. (2019). In silico identification and design of potent peptide inhibitors against PDZ-3 domain of Postsynaptic Density Protein (PSD-95). J. Biomol. Struct. Dyn..

[51] Sharma T., Harioudh M.K., Kuldeep J., Kumar S., Banerjee D., Ghosh J.K., Siddiqi M.I. (2020). Identification of Potential Inhibitors of Cathepsin-B using Shape & Pharmacophore-based Virtual Screening, Molecular Docking and Explicit Water Thermodynamics. Mol. Inform..

[52] Mothay D., Ramesh K.V. (2020). Binding Site Analysis of Potential Protease Inhibitors of COVID-19 Using AutoDock. VirusDisease.

[53] Forli S., Huey R., Pique M.E., Sanner M.F., Goodsell D.S., Olson A.J. (2016). Computational Protein-Ligand Docking and Virtual Drug Screening with the AutoDock Suite. Nat. Protoc..

[54] Goga N., Marin I., VasilǍţeanu A., PǍvǍloiu I.B., Kadiri K.O., Awodele O. Improved GROMACS Algorithms Using the MPI Parallelization. Proceedings of the 2015 E-Health and Bioengineering Conference (EHB).

[55] MacKerell A.D., Banavali N., Foloppe N. (2000). Development and Current Status of the CHARMM Force Field for Nucleic Acids. Biopolymers.

[56] Boonstra S., Onck P.R., van der Giessen E. (2016). CHARMM TIP3P Water Model Suppresses Peptide Folding by Solvating the Unfolded State. J. Phys. Chem. B.

[57] Laskowski R.A., Swindells M.B. (2011). LigPlot+: Multiple Ligand-Protein Interaction Diagrams for Drug Discovery. J. Chem. Inf. Model..

[58] Hubbard S.J., Thornton J.M. (1993). NACCESS.

[59] Schyman P., Liu R., Desai V., Wallqvist A. (2017). VNN Web Server for ADMET Predictions. Front. Pharmacol..

[60] Delaney J.S. (2004). ESOL: Estimating Aqueous Solubility Directly from Molecular Structure. J. Chem. Inf. Comput. Sci..

[61] Ali J., Camilleri P., Brown M.B., Hutt A.J., Kirton S.B. (2012). Revisiting the General Solubility Equation: In Silico Prediction of Aqueous Solubility Incorporating the Effect of Topographical Polar Surface Area. J. Chem. Inf. Model..

[62] Baell J.B., Holloway G.A. (2010). New Substructure Filters for Removal of Pan Assay Interference Compounds (PAINS) from Screening Libraries and for Their Exclusion in Bioassays. J. Med. Chem..

