# Supplementary material for: Nobiletin as a Neuroprotectant against NMDA Receptors: An In Silico Approach
Source: Pharmaceutics. 2022 May 25;14(6):1123. doi: 10.3390/pharmaceutics14061123 (PMC9229780; doi:10.3390/pharmaceutics14061123)
Supplement: Supplementary file 1 [file pharmaceutics-14-01123-s001.zip › pharmaceutics-1679063-supplementary.pdf]

Table S1. The analysis of NMDA proteins and their isoforms.

Name	Molecular Weights	GRAVY index	Instability index	Aliphatic index
GRIN2A isoform 1 (GLUN2A)	165282.52	−0.393	42.26	76.73
GRIN2B (GLUN2B)	166367.24	−0.388	48.76	74.55
GRIN2C (GLUN2C)	134208.94	−0.060	49.19	84.16
GRIN2D (GLUN2D)	143752.21	−0.192	57.34	77.78

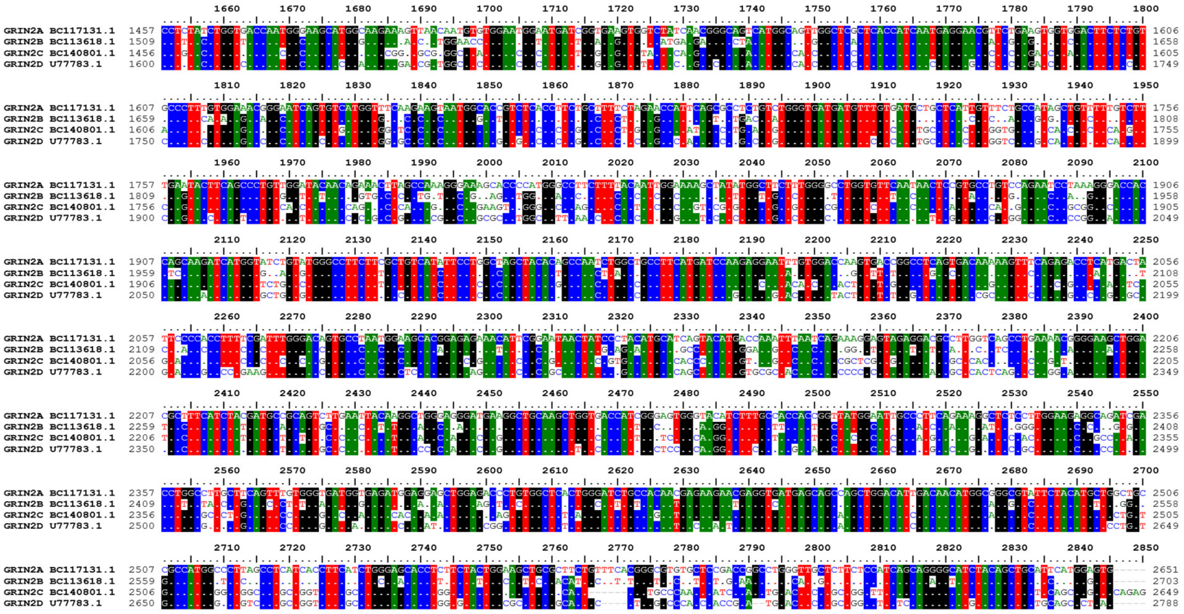

Figure S1. Multiple sequence alignment mRNA/coding sequence of GRIN2A, GRIN2B, GRIN2C AND GRIND2D genes.

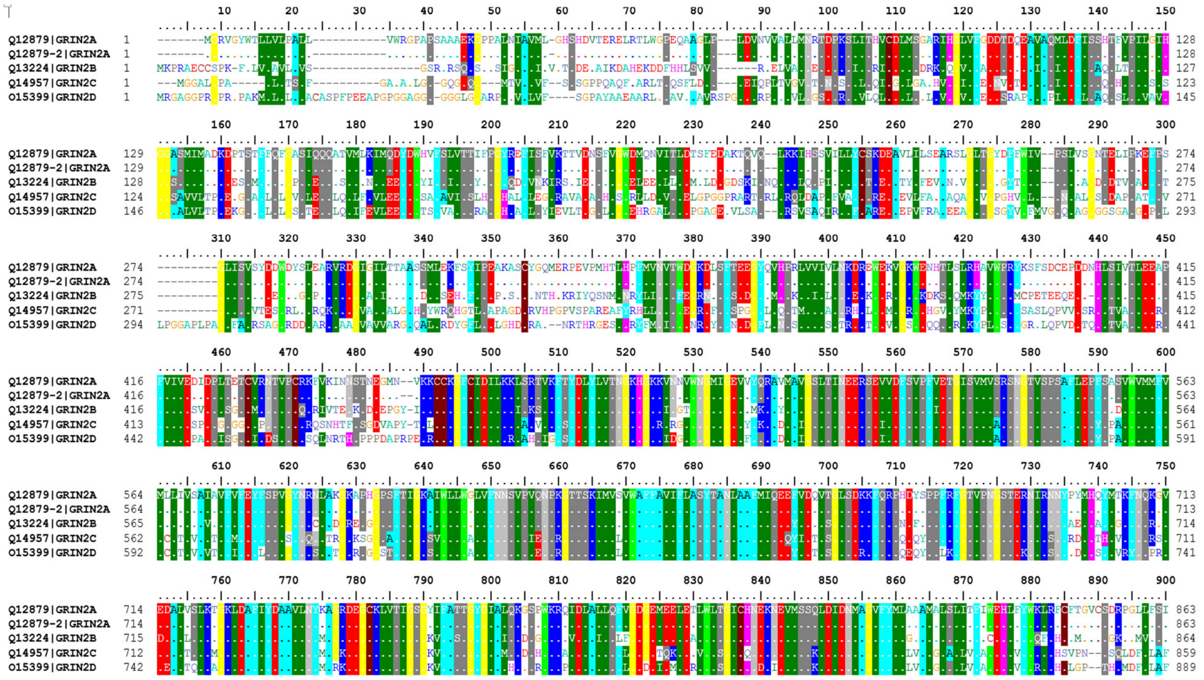

Figure S2. Multiple sequence alignment protein sequences are encoded by GRIN2A, GRIN2B, GRIN2C, and GRIND2D genes.

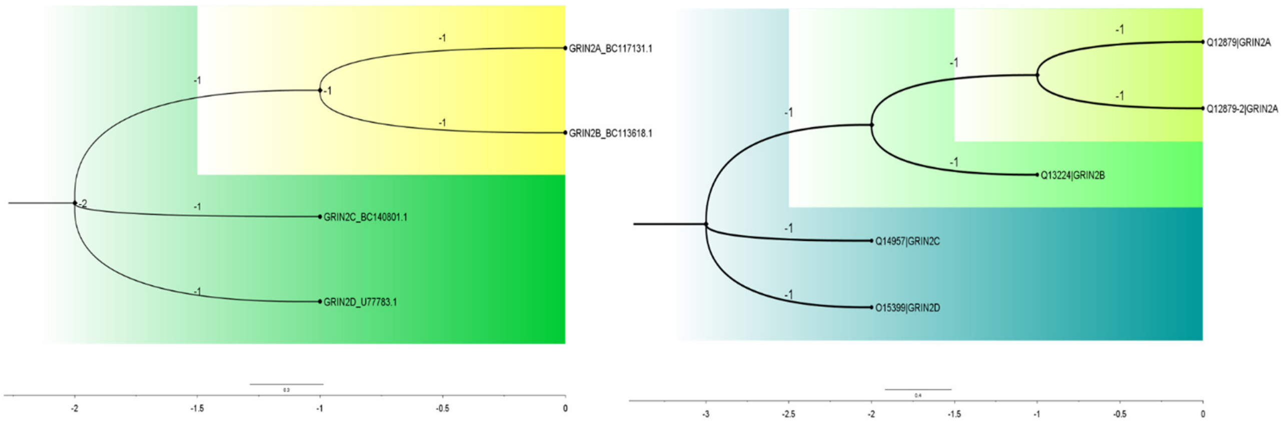

a

b

Figure S3. Phylogenetics tree created among (**a**) nucleotide sequence (**b**) protein sequence.
